# Home-based, supervised, and mixed exercise intervention on functional capacity and quality of life of colorectal cancer patients: a meta-analysis

**DOI:** 10.1038/s41598-022-06165-z

**Published:** 2022-02-15

**Authors:** Mauricio Beitia Kraemer, Denise Gonçalves Priolli, Ivan Gustavo Masseli Reis, Andrea Corazzi Pelosi, Ana Luíza Paula Garbuio, Leonardo Henrique Dalcheco Messias

**Affiliations:** Laboratory of Multidisciplinary Research, University of São Francisco, São Francisco de Assis Av, 218, Taboão, Bragança Paulista, São Paulo 12900000 Brazil

**Keywords:** Colorectal cancer, Cancer prevention

## Abstract

This systematic review and meta-analysis of randomized controlled trials tested the effects of home-based, supervised, or mixed exercise interventions on the functional capacity (FC) and quality of life (QoL) in colorectal cancer patients. A literature search was performed using the PubMed, Embase, Cochrane, and Medline databases. Two reviewers screened the literature through March 10, 2021 for studies related to exercise and colorectal cancer. Of the 1161 screened studies in the initial search, 13 studies met the eligibility criteria (home-based = 6 studies; supervised or mixed = 7 studies). Overall, 706 patients were enrolled in the trials, and 372 patients were submitted to home-based, supervised, or mixed exercise intervention. The overall results from the main meta-analysis showed a significant effect regarding supervised or mixed intervention (6 studies; p = 0.002; I^2^ = 43%; PI 0.41–1.39); however, no significant effect was observed for home-based intervention (5 studies; p = 0.05; I^2^ = 25%; PI − 0.34–0.76). A sensitivity analysis based on studies with intervention adherence ≥ 80% (home-based = 3 studies; supervised or mixed = 4 studies) revealed that home-based intervention or intervention entirely supervised or with some level of supervision (mixed) are effective in improving the QoL and FC of CRC patients. In summary, this meta-analysis verified that supervised and home-based exercise can modify QoL and FC when intervention adherence ≥ 80%. Regardless of the supervision characteristics, future RCTs are strongly encouraged to provide a detailed description of the exercise variables in physical interventions for CRC prescription. This perspective will allow a refined exercise prescription for patients with CRC, mainly according to their clinical status.

## Introduction

The incidence of colorectal cancer (CRC) cases globally increased by 9.5% between 1990 and 2017^1^. The American Cancer Society estimates 150,000 new CRC cases (colon—104,270; rectal—45,230) for 2021, only in the US^2^; and its burden across the world is expected to increase by approximately 2.2 million new cases and 1.1 million deaths by 2030^3^. Poor adherence to a healthy lifestyle, such as obesity, diabetes, smoking, alcohol, and a low-fiber high-fat diet are the risk factors for CRC incidence^4–6^. Moreover, physical inactivity and poor adherence to exercise training have also gained attention from the scientific community as being the relevant factors for the development of this disease^7^.

Regular physical exercise is recommended to prevent CRC^8^ and has been associated with reduced CRC-specific and all-cause mortality^9^. Systematic reviews of randomized controlled trials (RCTs), control trials, and cohort studies have shed light on the effects of physical exercise in patients with CRC. Gao et al.^10^ concluded that physical exercise improves aerobic power, metabolism, and tumor-related biomarkers in post-treatment CRC survivors. Reports included in the study by van Rooijen et al.^11^ consistently demonstrated that combined strength and endurance/interval physical training seems to be effective for improving the peak oxygen consumption and muscle strength during CRC treatment. Additionally, preoperative exercises may improve both the physical and functional fitness of CRC patients^12^.

The benefits of physical exercise for CRC patients are well studied; however, there is no concise information regarding the optimal exercise mode, frequency, duration, and intensity, even though the issue has been highlighted approximately 20 years ago^13^. The systematic review and meta-analysis of Singh et al.^14^ concluded that physical exercise following CRC diagnosis has a low risk of adverse events is feasible and provides health benefits irrespective of the exercise mode, duration, and level of supervision. Singh et al.^14^ initiated this discussion, but also included RCTs that tested exercise along with other interventions (e.g., nutritional or psychological) for CRC patients. This is notable and relevant given the emergent interest of researchers in multimodal approaches for CRC treatment^15–17^. Nevertheless, to solely discuss the effects of supervised and home-based approaches, we are particularly interested in RCTs that only conducted stand-alone exercise interventions for CRC patients. Thus, the analysis is restricted to the level of supervision, both home-based and supervised (or mixed) exercise interventions recurrently tested in RCTs involving CRC patients.

The benefits versus limitations of supervised versus home-based exercise has been a topic of debate^18–22^. CRC profoundly affects health and causes detrimental effects on the quality of life (QoL) and the functional capacity (FC)^23^. The QoL can be measured by a myriad of tools; however, the studies focused on cancer patients largely consider scores from validated questionnaires. The FC reflects the ability to perform regular activities by integrating the cardiovascular, pulmonary, and skeletal muscle systems largely under aerobic conditions^24^. There are potential advantages and disadvantages to supervised versus home-based exercise; however, currently, no review has attempted to compare the sole effect of these interventions on the QoL and FC in CRC patients. We aimed to compare the effects of supervised/mixed versus home-based interventions on QoL and FC of CRC patients. Secondarily, we aimed to perform a sensitivity analysis by only comparing exercise interventions with high adherence on QoL and FC.

## Methods

### Search strategy

The literature search was performed through March 10, 2021 in the PubMed, Embase, Cochrane, and Medline databases with no restrictions with respect to time. The Boolean operators “AND”, “OR”, and “NOT” were adopted to key terms, such as “colorectal neoplasm OR colorectal cancer” AND “resistance training OR endurance training OR high-intensity interval training OR plyometric training OR exercise.” The filters for “RCT” and “humans” were activated during the searches. A manual search of the reference lists and citations from articles related to CRC was also performed.

### Eligibility criteria

The Preferred Reporting Items for Systematic Reviews and Meta-Analyses (PRISMA)^25^ was used to screen the studies involving CRC patients. Additionally, the PICOS criteria^26^ (Table 1) were adopted as the eligibility criteria. Meticulous inclusion consisted of: (a) RCT, preliminary, or feasibility RCTs involving the exercise intervention in CRC patients and published in English; (b) studies involving interventions rather than an only association between the outcomes; (c) studies including other cancer types were considered as long as they provided results of the CRC subgroup; (d) the only intervention consisted of exercise, which was structured and provided detailed information on the intensity or duration; (e) the length of the intervention (e.g. number of weeks) and type (i.e. home-based, supervised, or mixed) was clearly presented; (f) studies that described the exercises performed by patients (e.g. running, walking, cycling); and (g) the outcomes are related to the QoL (e.g. global scores from validated QoL questionnaires, or scores from other questionnaires focused on the parameters that directly affect the QoL of CRC patients, such as fatigue, depression, anxiety, sleep), and/or FC (e.g. measures of aerobic power—peak oxygen uptake—or capacity—anaerobic threshold, cardiovascular responses, such as the heart rate, total distance covered, and time to exhaustion of physical evaluations). The meticulous exclusion consisted of: (a) studies with animals; (b) grey literature without detailed information, data, or full text; (c) abstracts without full-text; (d) lack of detailed information on the exercises, duration, intensity, or the length of intervention; and (e) studies that did not include pre-post statistical analysis.

**Table 1:** Eligibility criteria for inclusion of studies in this systematic review according to the PICOS (population, intervention, comparator, outcomes, and study design) criteria.

Components	Feature
Population	CRC patients
Intervention	Home-based, supervised, or mixed physical exercise interventions
Comparator	CRC patients not enrolled in physical exercise intervention
Outcomes	Quality of life and functional capacity measures
Study design	Randomized-controlled trials

*CRC* colorectal cancer.

### Data extraction

Two reviewers (LM and AG) independently screened the studies based on the PICOS and meticulous inclusion/exclusion. The discrepancies were checked by a third reviewer (IR), and a consensus was reached through discussion. Further data on the strength or resistance outcomes were not shown and demonstrated since we were particularly interested in the effects of home-based, supervised, or mixed exercise interventions on the QoL and FC. The follow-up results were not presented or discussed according to the goals of this systematic review.

### Quality assessment

The quality analysis of the included studies was performed independently by two reviewers (LM and AG) according to the Physiotherapy Evidence Database (PEDro) scale^27^. PEDro is composed of 11 items that yield one point (except for the first item) to the final score and its reliability of items. Additionally, the final score varied from fair to substantial and fair to good, respectively^28^.

### Meta-analysis of QoL and FC

The quantitative data related to the QoL and FC from home-based or supervised exercise interventions were extracted for the meta-analysis and considered as continuous variables. Given that mixed approaches have some level of supervision, we considered these results as supervised interventions for the meta-analysis. A sensitivity analysis was conducted with high intervention adherence and retention rate (≥ 80%). The ≥ 80% was considered as high adherence based on previous suggestions with cancer patients^29–31^. Adherence was defined as the number of sessions attended out of total offered. Two studies did not report exercise attendance^32,33^, but informed that the data from patients with low adherence to exercise was not included in the analysis. In these cases, the retention rate (i.e. number of patients that completed the exercise intervention out of total patients allocated to the exercise group) was considered instead of the attendance. The post-intervention means and standard deviations (SDs) were used for comparisons. The effect size index was considered as the standardized mean difference (SMD) and was used for the meta-analysis with 95% confidence intervals (CI). A random effects model was used for the analysis. The Z-value, I^2^, and prediction interval (PI) were adopted for significance analysis, proportion estimation of variance, and variation in the treatment effects, respectively. SMD was plotted against the standard error, and funnel plots were generated to evaluate the asymmetries around the metanalytic summary effect^34^. Two studies were excluded from the meta-analysis because they did not present the standard deviation^31,35^. In three studies^33,36,37^ the inferences of the results were the opposite. This means that as the parameter decreases, the outcome is observed to improve. In such cases, the minus sign was inserted into the means of both the intervention and control values. All statistical analyses, except PI, were performed using the Review Manager 5 software (version 5.3, The Nordic Cochrane Centre, Copenhagen, Denmark). The PI was calculated using a comprehensive meta-analysis software (CMA)^38^. The statistical significance was set at p < 0.05.

## Results

### Literature search and quality assessment

**Figure 1:**
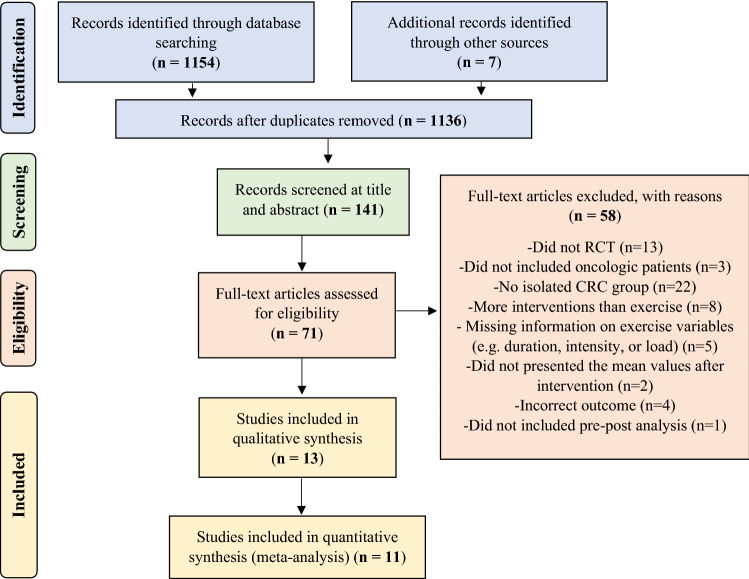
Flow diagram of the preferred reporting items for systematic reviews and meta-analyses (PRISMA).

The initial literature search identified 1161 studies (Fig. 1). Based on the first search, 25 duplicates were identified. After screening for the title, abstract, and full text, 13 studies^17,31–33,35–37,39–44^ attained the eligibility criteria (Table 2). The included studies were published between the years 2003 and 2020. The mean and standard deviation of the PEDro score among the studies was 6.46 ± 0.92. Three studies were classified with a score of 8, one with a score of 7, eight studies with a score of 6, and one study with a score of 5.

**Table 2:** Overview of the included studies.

Study	Sample size and age	Patients’ status and eligibility criteria	Stage of cancer	Tumor location
Courneya et al. (2003)^41^	*Control* n = 31 (M = 20; F = 11); age = 61 ± 13 year *Intervention* n = 62 (M = 34; F = 28); age = 59 ± 10 year	Patients who had surgery for CRC within the past 3 months and have recovered from it	I–II (n = 4) III (n = 27) I–II (n = 14); III (n = 44); IV (n = 4)	Colon (74.2%) Rectum (25.8%) Colon (77%) Rectum (23%)
Ahn et al. (2013)^36^	*Control* n = 14 (M = 5; F = 9); age = 57 ± 6 year *Intervention* n = 17 (M = 12; F = 5); age = 55 ± 7 year	Patients diagnosed with stages I–III of CRC	I (n = 3); II (n = 6); III (n = 5) I (n = 7); II (n = 5); III (n = 5)	Ascending (43%) Transverse (7%) Descending (7%) Sigmoid (0%) Rectosigmoid (43%) Splenic Flexure (0%) Ascending (24%) Transverse (6%) Descending (0%) Sigmoid (6%) Rectosigmoid (53%) Splenic Flexure (12%)
Pinto et al. (2013)^35^	*Control* n = 26 (M = 12; F = 14); age = 55 ± 8 year *Intervention* n = 20 (M = 8; F = 12); age = 59 ± 11 year	Sedentary patients who completed (≤ 5 year) primary and adjuvant treatments for stages I–III of CRC	Patients who completed primary and adjuvant treatments for colon or rectal cancer (stages I–III)	Colon (58%) Rectum (42%) Colon (55%) Rectum (45%)
Lin et al. (2014)^44^	*Control* n = 24 (M = 15; F = 9); age = 54 ± 10 year *Intervention* n = 21 (M = 11; F = 10); age = 59 ± 9 year	Patients diagnosed with stages II–III of CRC, had undergone elective, abdominal colorectal surgery and had ECOG performance less than 3	IIA (n = 6); IIB (n = 0); IIIA (n = 4); IIIB (n = 10); IIIC (n = 4) IIA (n = 4); IIB (n = 4); IIIA (n = 2); IIIB (n = 4); IIIC (n = 7)	Not specified
Cantarero-Villanueva et al. (2016)^39^	*Control* n = 19 (M = 13; F = 6); age = 62 ± 7 year *Intervention* n = 21 (M = 13; F = 8); age = 57 ± 5 yr	Patients who received curative treatment due to cancer and completed coadjuvant treatment	II (n = 7); IIIa (n = 12) II (n = 7); IIIa (n = 14)	Not specified
Lee et al. (2017)^42^	*Control* n = 61 (M = 28; F = 33); age = 56 ± 9 years *Intervention* n = 62 (M = 31; F = 31); age = 56 ± 9 years	Patients diagnosed with stages II–III of CRC, completed surgery, radiotherapy, and/or chemotherapy within four weeks to two years before study enrollment, and ECOG performance status of 0 or 1	II (n = 23); III (n = 38) II (n = 34); III (n = 27)	Colon (65.6%) Rectum (34.4%) Colon (72.1%) Rectum (27.9%)
Zimmer et al. (2018)^17^	*Control* n = 13 (M = 9; F = 4); age = 70 years^a^ *Intervention* n = 17 (M = 12; F = 5); age = 68 years^a^	Patients with metastasized CRC and estimated life expectancy of at least 6 months	IV (n = 13) IV (n = 17)	Cecum/Colon (52.9%) Rectosigmoid (11.8%) Rectum (35.3%) Cecum/Colon (61.5%) Rectosigmoid (7.7%) Rectum (30.8%)
Lee et al. (2018)^43^	*Control* n = 34 (M = 17; F = 17); age = 56 ± 10 years *Intervention* n = 38 (M = 18; F = 20); age = 55 ± 8 years	Patients diagnosed with stages II—III of CRC, completed surgery radiotherapy, and/or chemotherapy within four weeks to two years before study enrollment	II (n = 12); III (n = 22) II (n = 21); III (n = 17)	Colon (55.9%) Rectum (44.1%) Colon (71.1%) Rectum (28.9%)
Kim et al. (2019)^32^	*Control* n = 34 (M = 17; F = 17); age = 56 ± 10 years *Intervention* n = 37 (M = 18; F = 19); age = 55 ± 8 years	Patients diagnosed with stages II–III of CRC, completed surgery radiotherapy, and/or chemotherapy within 4 weeks to 2 years before study enrollment and ECOG performance status of 0 or 1	II (n = 12); III (n = 22) II (n = 21); III (n = 16)	Colon (73%) Rectum (27%) Colon (55.9%) Rectum (44.1%
Lu et al. (2019)^33^	*Control* n = 44 (M = 30; F = 14); age = 54 ± 11 year *Intervention* n = 43 (M = 26; F = 17); age = 55 ± 11 year	Patients undergoing chemotherapy diagnosed with stages I–III of CRC and had surgical resection of gastrointestinal tumors by laparotomy or laparoscopy under general anesthesia	I (n = 13); II (n = 26); III (n = 5) I (n = 10); II (n = 29); III (n = 4)	Colon (36%) Rectum (64%) Colon (42%) Rectum (58%)
Christensen et al. (2019)^40^	*Control* n = 20 (M = 11; F = 9); age = 60 ± 8 years *Intervention* n = 19 (M = 7; F = 12); age = 57 ± 10 year	Patients with CRC who had completed surgery for local stage disease (UICC stage I to IIa) and patients who had completed surgery and any adjuvant chemotherapy for locally advanced-stage disease (UICC stage IIb to III)	I (n = 4); II (n = 7); III (n = 9) I (n = 6); II (n = 4); III (n = 9)	Colon (85%) Rectum (15%) Colon (74%) Rectum (26%)
Karlsson et al. (2019)^31^	*Control* n = 11 (M = 4; F = 7); age = 74 years^b^ *Intervention* n = 10 (M = 4; F = 6); age = 83 years^b^	Patients scheduled for surgery due to CRC or suspected CRC	0 (n = 2); I (n = 3); II (n = 1); III (n = 5) II (n = 5); III (n = 4); IV (n = 1)	Colon (82%) Rectum (18%) Colon (90%) Rectum (10%)
Hwang et al. (2020)^37^	*Control* n = 3 (M = 1; F = 2); age = 62 ± 13 years *Intervention* n = 5 (M = 2; F = 3); age = 54 ± 10 year	Patients diagnosed with stages II–IV of CRC, underwent surgery for the primary treatment completed adjuvant chemotherapy or radiotherapy at least 5 years before study participation and ECOG performance status of 0–2	I–IV (not specified by stage or group)	Colon (62.5%) Rectum (37.5%) Colon (60%) Rectum (40%)

Note that only the most important eligibility criteria of studies (according to this systematic review goals) were included; ^a^variation not specified; ^b^median.

*M* male, *F* female, *ECOG* Eastern Cooperative Oncology Group, *UICC* Union for International Cancer Control.

### Patients and intervention characteristics

Overall, 706 patients with CRC were enrolled in the RCTs involving physical exercise as an intervention. Among these, 334 patients were controls, and 372 patients were submitted to home-based, supervised, or mixed exercise interventions. Only two studies included CRC patients with metastasis^17,41^. Home-based interventions occurred in six studies, while supervised and mixed interventions occurred in four and three studies, respectively. Five studies evaluated both outcomes^17,35,40,41,44^, and two studies^32,33^ only evaluated the QoL, and the remaining six studies evaluated the FC^31–33,39,42,43^. The majority of the included RCTs (85%) specified the cancer type and two studies^17,36^ provided further information on the tumor location.

### Instruments for quality of life assessment

The Functional Assessment of Cancer Therapy-Colorectal questionnaire (FACT-C)^45^ was adopted in four studies^32,35,40,41^. Further questionnaires, such as the Standard Chinese version of the European Organization for Research and Treatment of Cancer Quality of Life Core Questionnaire (EORTC QLQ-C30)^46^ and the Trial Outcome Index (TOI) were applied in one^44^ and three^17,32,40^ studies, respectively. One study^33^ used the Brief Fatigue Inventory (BFI) since fatigue profoundly impacts the QoL of CRC patients^47^. Two studies^32,40^ evaluated the QoL using both the FACT-C and TOI approaches.

### Instruments for functional capacity assessment

Six studies^17,31,39,42–44^ assessed the FC using the 6 Minute Walk Test (6-MWT), while two^36,43^ studies adopted the Tecumseh test and the Modified Balk Treadmill and TreadWalk tests were conducted in the studies of Courneya et al.^41^ and Pinto et al.^35^ respectively. Lee et al.^43^ assessed the FC using both 6-MWT and Tecumseh protocols.

### Effects of home-based exercise intervention on quality of life and functional capacity

Table 3 shows the RCTs that conducted home-based exercise interventions for CRC patients. The duration of the intervention ranged from 6 to 16 weeks. Two studies^35,41^ reported the prescribed exercise session frequency (i.e., days per week). The remaining studies used the metabolic equivalent tasks (MET) per hour^32,42,43^ and the amount of minutes per week^40^ to classify the frequency, and details on the exercise distribution over a week were not provided. Christensen et al.^40^ instructed the patients to perform exercises for 150 min/week according to their preferences. Likewise, Lee et al.^43^ submitted CRC patients to home-based exercises of more than 18 METs hours per week, which should count for approximately 360 min/week. While two studies applied an upper limit of 30-min daily exercise session duration^35,41^, others used the number of steps as a goal (i.e., > 10.000)^32,42,43^. With one exception^40^, all the studies prescribed the intensity based on a percentage (range 64–76%) of the maximum heart rate (HR_max_). Two studies applied only aerobic exercises^40,41^, and the remaining conducted both aerobic and resistance exercises^32,35,42,43^.

**Table 3:** Studies design and Quality of Life (QoL) and Functional Capacity (FC) outcomes of home-based exercise interventions in colorectal patients.

Study	Length of intervention (L) (weeks) Frequency (F) (weekly) Exercise adherence (A) (%)	Exercise session duration (D) Intensity (I)	Exercises	Instrument for QoL measurement (parameter)	QoL results	Instrument for FC measurement (parameter)	FC results
Courneya et al. (2003)^41^	L = 16 F = 3–5 A: 75.8	D = 20–30 min I = 65–75% of the HR_max_	Swimming, cycling or walking	FACT-C (score)	*Control* Pre = 107.0 ± 16.0 Pos = 109.8 ± 18.8 *Intervention* Pre = 106.0 ± 14.0 Pos = 107.4 ± 16.5	Modified Balk Treadmill Test (time spent in the test)	*Control* Pre = 314 ± 270 s Pos = 406 ± 301 s *Intervention* Pre = 396 ± 291 s Pos = 548 ± 300 s
Pinto et al. (2013)^35^	L = 12 F = 2–5 A: 76	D = 10–30 min I = 64–76% of the HR_max_	Brisk walking, biking, or use of home exercise equipment	FACT-C (score)	*Control* Pre = 105.3 Pos = 110.8 *Intervention* Pre = 105.3 Pos = 111.3	Treadwalk test (VO2_peak_)	*Control* Pre = 22.9 ml/kg/min Pos = 23.7 ml/kg/min *Intervention* Pre = 22.9 ml/kg/min Pre = 27.6 ml/kg/min^a^
Lee et al. (2017)^42^	L = 12 F = First 6 weeks–> 18 MET-hours A: 86.3 F = After 6th week–27 MET-hours A: 74.5 Mean total adherence: 80.4	D = > 10,000 steps plus 30 min of resistance exercises using the body weight I = Of the 10.000 steps, 3.000 should be up to 65% of the HR_max_	Brisk walking, hiking, stationary bike, and resistance exercises with own body mass	NA	NA	6-MWT (distance covered)	*Control* Pre = 598 ± 75 m Pos = 588 ± 72 m *Intervention* Pre = 578 ± 79 m Pos = 603 ± 74 m^a^
Lee et al. (2018)^43^	L = 6 F = > 18 MET-hours A: 73.5	D = > 10,000 steps plus 30 min of resistance exercises using the body weight I = Of the 10.000 steps, 3.000 should be up to 65% of the HR_max_	Brisk walking, hiking, stationary bike, and resistance exercises with own body mass	NA	NA	Tecumseh test (HR measured 1 min after the test) 6-MWT (distance covered)	Tecumseh—*Control* Pre = 93 ± 14 bpm Pos = 95 ± 13 bpm Tecumseh—*Intervention* Pre = 92 ± 13 bpm Pos = 88 ± 13 bpm^a^ 6-MWT—*Control* Pre = 582 ± 70 m Pos = 594 ± 96 m 6-MWT—*Intervention* Pre = 576 ± 85 m Pos = 585 ± 82 m
Kim et al. (2019)^32^	L = 12 F = First 6 weeks—> 18 MET-hours F = After 6th week—27 MET-hours A: 81.1^c^	D = > 10,000 steps plus 30 min of resistance exercises using the body weight I = Of the 10.000 steps, 3.000 should be up to 65% of the HR_max_	Brisk walking, hiking, stationary bike, and resistance exercises with own body mass	TOI (score) FACT-C (score)	TOI—*Control* Pre = 63.4 ± 13.1 Pos = 64.3 ± 12.4 TOI—*Intervention* Pre = 64.1 ± 11.2 Pos = 66.3 ± 11.8^a^ FACT-C—*Control* Pre = 97.5 ± 19.9 Pos = 99.1 ± 19.1 FACT-C—*Intervention* Pre = 100.5 ± 18.1 Pos = 104.3 ± 17.5^a^	NA	NA
Christensen et al. (2019)^40^	L = 12 F = 150 min/week performed according to the patient’s preferences A: Training logs = 102 ± 16 A: InterWalk = 79 ± 10 Mean total adherence: 90.5^b^	Not specified	Walking	TOI (score) FACT-C (score)	TOI—*Control* Pre = 66.4 ± 12.7 Pos = 68.4 ± 10.2 TOI—*Intervention* Pre = 68.0 ± 11.6 Pos = 73.8 ± 7.6^a^ FACT-C—*Control* Pre = 108 ± 19 Pos = 110 ± 18 FACT-C—*Intervention* Pre = 112 ± 17 Pos = 121 ± 11^a^	Incremental test (VO2_peak_)	*Control* Pre = 26.1 ± 6.0 ml/kg/min Pos = 24.8 ± 5.7 ml/kg/min *Intervention* Pre = 26.9 ± 6.9 ml/kg/min Pos = 26.4 ± 5.7 ml/kg/min

*HRmax* maximum heart rate, *FACT-C* functional assessment of cancer therapy-colorectal, *VO2*_*peak*_ peak oxygen uptake, *6-MWT* 6 Minute Walk Test, *TOI-PEC* trial outcome index-physical/functional/colorectal.

^a^Denote difference from Pre.

^b^The adherence was obtained by training logs and an application named InterWalk.

^c^Retention rate.

**Figure 2:**
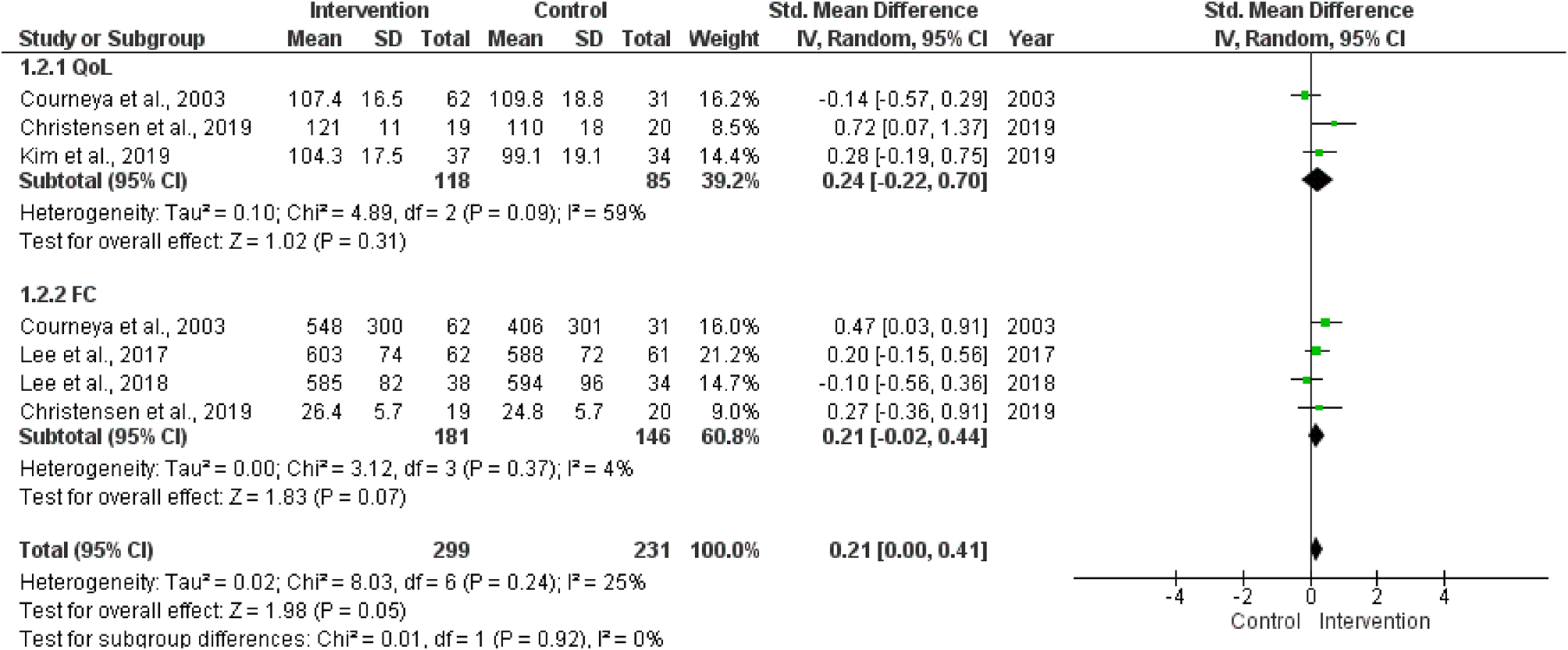
Meta-analysis of quality of life (QoL) and functional capacity (FC) measured in randomized controlled trials that conducted home-based exercise intervention for colorectal cancer patients.

**Figure 3:**
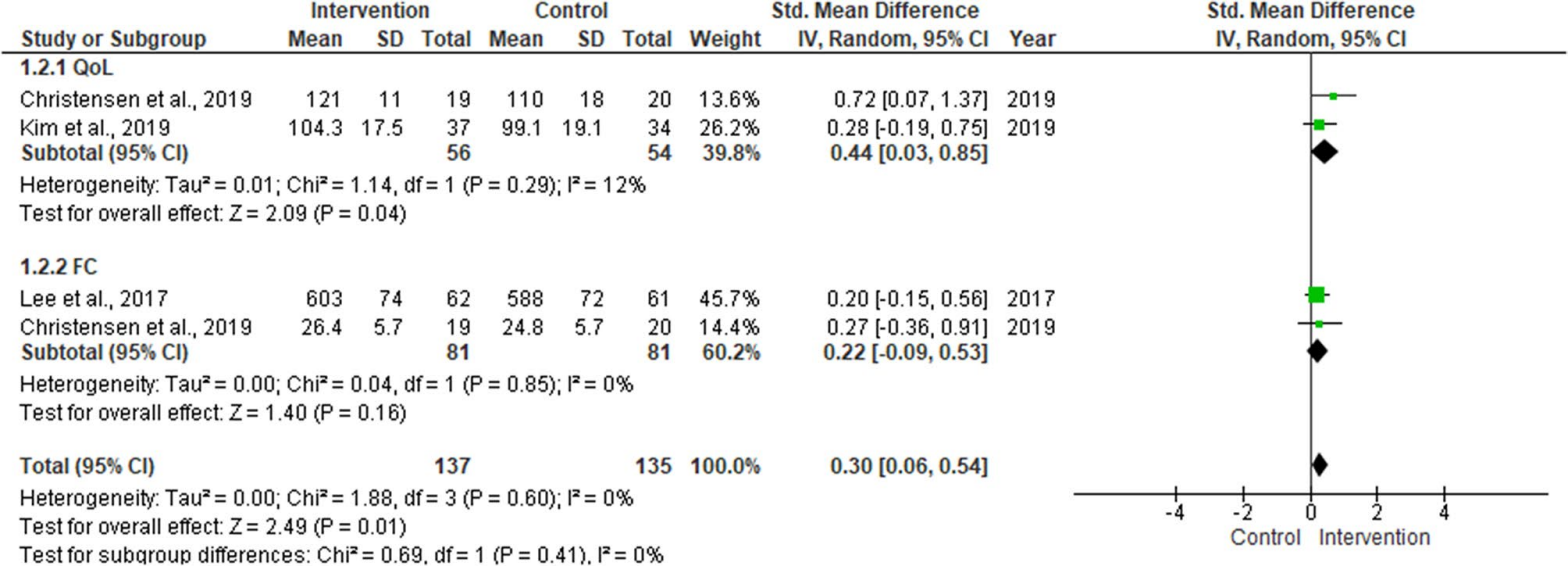
Sensitivity analysis of quality of life (QoL) and functional capacity (FC) measured in randomized controlled trials that conducted home-based exercise intervention with ≥ 80% adherence for colorectal cancer patients.

A meta-analysis of the QoL and FC is shown in Fig. 2. No significant effect of home-based exercise on QoL and a considerable proportion of variance were observed. Further, the PI of QoL was − 4.76–5.24. A low proportion of variance was observed for FC and the significance was almost reached; besides, CMS returned that all studies share a common effect size. Overall, the significance was observed at p = 0.05, with a low proportion of variance and a PI of − 0.34–0.76. The mean adherence to home-based interventions was observed at 80.2 ± 6.1%. The sensitivity analysis included three studies^32,40,42^ and revealed a significant effect of home-based intervention regardless of the outcome (overall, p = 0.01; I^2^ = 0%) (Fig. 3).

### Effects of supervised or mixed exercise intervention on the quality of life and functional capacity

The characteristics and outcomes of the three studies that conducted supervised exercise interventions in CRC patients are shown in Table 4. The length of the intervention ranged from 8 to 24 weeks, with 2^17,44^, 4^39^, or 5^33^ weekly exercise session frequency. The upper limit of the exercise session duration of supervised intervention ranged from 40 to 90 min. The intensity of exercise was not specified in two studies^33,39^. The two remaining studies used the HR_max_ or rating of perceived exertion (RPE) scores for prescribing endurance activities. Additionally, Zimmer et al.^17^ prescribed resistance exercises based on the percentage of one maximum repetition (1RM) or Borg’s category ratio-scale (Borg CR10). Lin et al.^44^ and Zimmer et al.^17^ applied both endurance and strength exercises, while the intervention of Lu et al.^33^ was solely based on Baduanjin qigong. Cantarero-Villanueva et al.^39^ conducted aerobic and stabilization exercises.

**Table 4:** Studies design and Quality of Life (QoL) and Functional Capacity (FC) outcomes of supervised exercise interventions in colorectal patients.

Study	Length of intervention (P) (weeks) Frequency (F) (weekly) Exercise adherence (A) (%)	Exercise session duration (D) Intensity (I)	Exercises	Instrument for QoL measurement (parameter)	QoL results	Instrument for FC measurement (parameter)	FC results
Lin et al. (2014)^44^	L = 12 F = 2 A: 73	D = 40–60 min I = Initial—40–55% of the HR_max_ or 11–12 of RPE Progressed—60–75% of the HR_max_ or 14–15 of RPE	Aerobic—cycling Resistance—theraband, dumbbell, and sandbag	EORTC QLQ-C30 (score)	*Control* Pre = 68.1 ± 18.5 Pos = 72.2 ± 17.5 *Intervention* Pre = 67.9 ± 16.3 Pos = 71.4 ± 19.8	6-MWT (distance covered)	*Control* Pre = 505 ± 117 m Pos = 550 ± 121 m *Intervention* Pre = 491 ± 91 m Pos = 550 ± 85 m Effect of time p = 0.02
Cantarero-Villanueva et al. (2016)^39^	L = 8 F = 4 A: 88.3	D = 90 min I = not specified	Aerobic—Brisk walking or running Stabilization—pilates, including local segmental control exercise, roll up-roll down, one leg circle, side kicks, saw, hundred and leg pull front Stretching	NA	NA	6-MWT (distance covered)	*Control* Pre = 288 ± 132 m Pos = 293 ± 125 m *Intervention* Pre = 330 ± 137 m Pos = 410 ± 130 m Interaction p < 0.01
Zimmer et al. (2018)^17^	L = 8 F = 2 A: 80	D = 60 min I = Endurance training—60–70% of the HR_max_ or 12–13 of RPE Resistance training—60–80% of 1RM or 6 score at Borg CR10	Phase 1—Balancing and coordination practices Phase 2—cross-training, cycling, or walking (Endurance) and bench press, lat pulldown, leg press, seated row, and abdominal (Resistance) Phase 3—relaxing, stretching, breathing and mobilization	TOI (score)	*Control* Pre = 71.5 ± 13.0 Pos = 64.4 ± 11.5 *Intervention* Pre = 75.0 ± 14.8 Pos = 77.3 ± 11.8	6-MWT (distance covered)	*Control* Pre = 459 ± 74 m Pos = 478 ± 75 m *Intervention* Pre = 477 ± 91 m Pos = 502 ± 62 m
Lu et al. (2019)^33^	L = 24 F = 5 A: 95^a^	D = 20–40 min I = Not specified	Baduanjin qigong	BFI (score)	*Control* Pre = 4.7 ± 2.5 Pos 1 = 4.4 ± 2.4 Pos 2 = 4.1 ± 1.9 *Intervention* Pre = 4.4 ± 2.2 Pos 1 = 4.3 ± 2.1 Pos 2 = 2.7 ± 2.1 Effect of time p < 0.01 Interaction p < 0.01	NA	NA

The EORTC QLQ-C30 provides several outcomes related to the quality of life. Therefore, we opted to expose those who were modified by the intervention; Pos 1—reevaluation at the 12th week; Pos 2—reevaluation at the 24th.

*EORTC QLQ-C30* European Organization for Research and Treatment of Cancer Quality of Life Core Questionnaire, *6-MWT* 6 Minute Walk Test, *TOI* Trial Outcome Index, *RPE* Rating of perceived exertion, *HR*_*max*_ Maximum Heart Rate, *1RM* one maximum repetition, *Borg CR10* Borg's category ratio-scale, *BFI* Brief Fatigue Inventory.

^a^Retention rate.

Only one study fixed the length of mixed exercise intervention in CRC patients^37^. This approach was not possible in studies by Ahn et al.^36^ and Karlsson et al.^31^ since the main goals of these RCTs are directly associated with the intervention length. The exercise session duration ranged from 30 to 60 min in these studies. Additionally, only the RCT by Karlsson et al.^31^ prescribed an exercise intensity based on an inspiratory test (maximal inspiratory pressure [MIP]) or ratings from the Borg CR10. Two studies adopted both strength and aerobic exercises^31,36^ and Hwang et al.^37^ mostly prescribed resistance exercises. The QoL and FC did not improve in these studies (Table 5).

**Table 5:** Studies design and Quality of Life (QoL) and Functional Capacity (FC) outcomes of exercise interventions with some level of supervision (i.e. mixed) in colorectal patients.

Study	Length of intervention (P) (weeks) Frequency (F) (weekly) Exercise adherence (A) (%)	Exercise session duration (D) Intensity (I)	Exercises	Instrument for QOL measurement (parameter)	QOL results	Instrument for FC measurement (parameter)	FC results
Ahn et al. (2013)^36^	**L** = *Control*^a^ 9.8 ± 2.6 days Intervention^a^ 7.8 ± 1.0 days Phase 1—Twice/day supervised session plus unsupervised sitting or walking Phase 2—same as phase 1 Phase 3—One supervised and one unsupervised exercise session plus sitting or walking **F** = NA A: 84.5	**D** = 30 min Details of supervised and unsupervised session duration not specified **I** = Phase 1—Very low Phase 2 -1-lb Weight Phase 3 -not specified	Phase 1—Stretching, core, and resistance exercises (in bed) plus unsupervised sitting or walking in the ward Phase 2—Stretching, core, and resistance exercises (limited ambulation) plus unsupervised walking in the hallway Phase 3—phase 2 exercises plus balancing exercises	NA	NA	Tecumseh test (HR measured 1 min after the test)	*Control* Pre = 92 ± 14 bpm Pos = 100 ± 12 bpm *Intervention* Pre = 89 ± 12 bpm Pos = 90 ± 11 bpm
Karlsson et al. (2019)^31^	**L** = At least 2 weeks or until the surgery **F** = Supervised—2–3 Unsupervised—150 min/week performed according to the patient’s preferences A: > 80	**D = **Supervised—60 min Unsupervised—core set of strength exercised and 30 inspiratory repetitions **I = **Inspiratory—50% of maximal capacity at the beginning and progressed until the achievement of 5–7 at Borg CR10 Strength—7–8 at Borg CR10 Aerobic—7–8 at Borg CR10	Inspiratory exercise Chair stands and step-up (strength) Stair climbing, Nordic walking outdoors, and interval walking indoors and/or outdoors (aerobic)	NA	NA	6-MWT (distance covered)	*Control* Pre = 432 m Pos 1 = 426 m Pos 2 = 278 m *Intervention* Pre = 418 m Pos 1 = 398 m Pos 2 = 330 m
Hwang et al. (2020)^37^	**L** = 6 **F** = Supervised—1 Unsupervised—6 A: Not reported	**D** = Supervised—30 min Unsupervised—not specified **I** = Not specified	Push-up, squat, shoulder press, sit-ups, pelvic tilt, shoulder bridge, bird-dog, pelvic abduction, squeezing ball with knees, calf raise, and superman	NA	NA	Tecumseh test (HR measured 1 min after the test)	*Control* Pre = 76 ± 8 bpm Pos = 79 ± 11 bpm *Intervention* Pre = 79 ± 13 bpm Pos = 81 ± 9 bpm

The results of Karlsson et al.^31^ were expressed as median.

*HR* heart rate, *Borg CR10* Borg's category ratio-scale, *Pos 1* pre-surgery, *Pos 2* pos-surgery.

^a^The intervention and its length are directly related.

**Figure 4:**
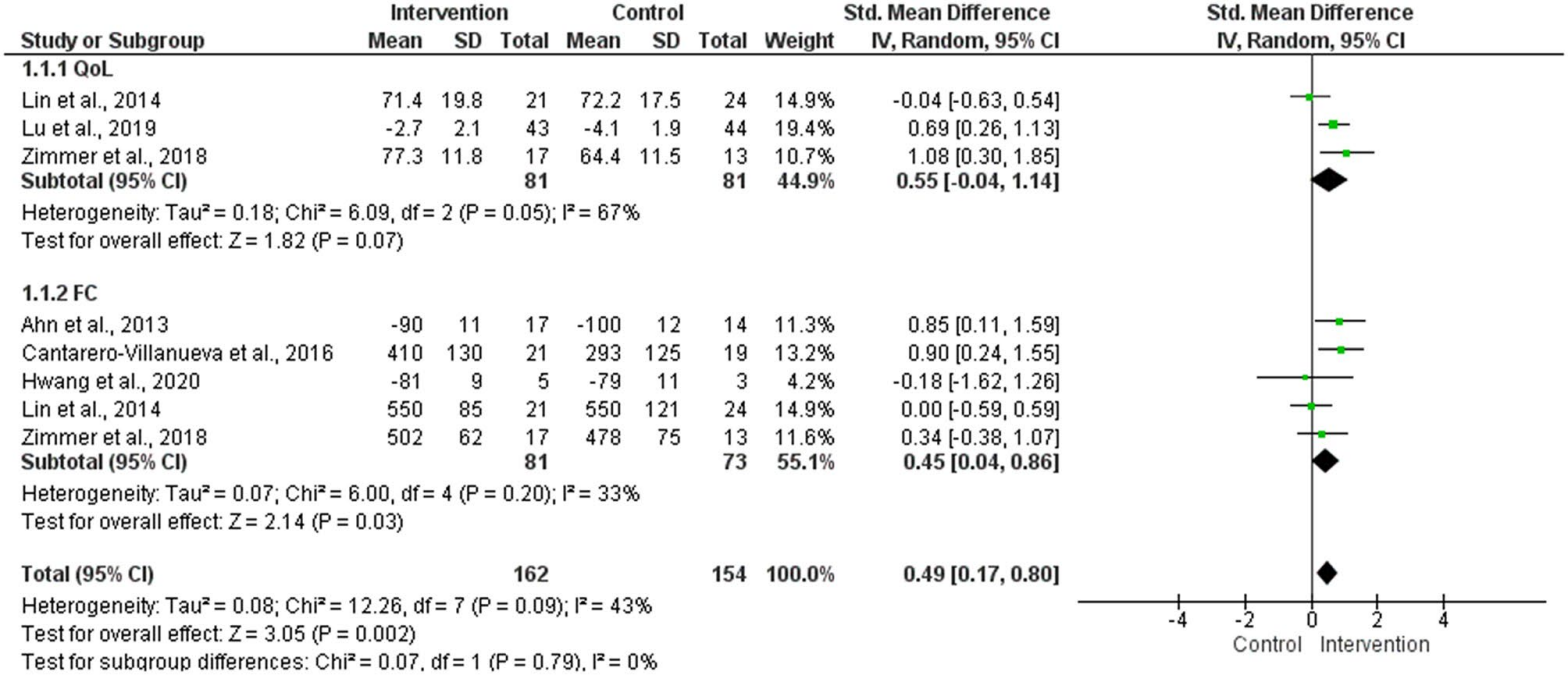
Meta-analysis of quality of life (QoL) and functional capacity (FC) measured in randomized controlled trials that conducted exercise intervention entirely supervised or with some level of supervision (mixed) for colorectal cancer patients.

**Figure 5:**
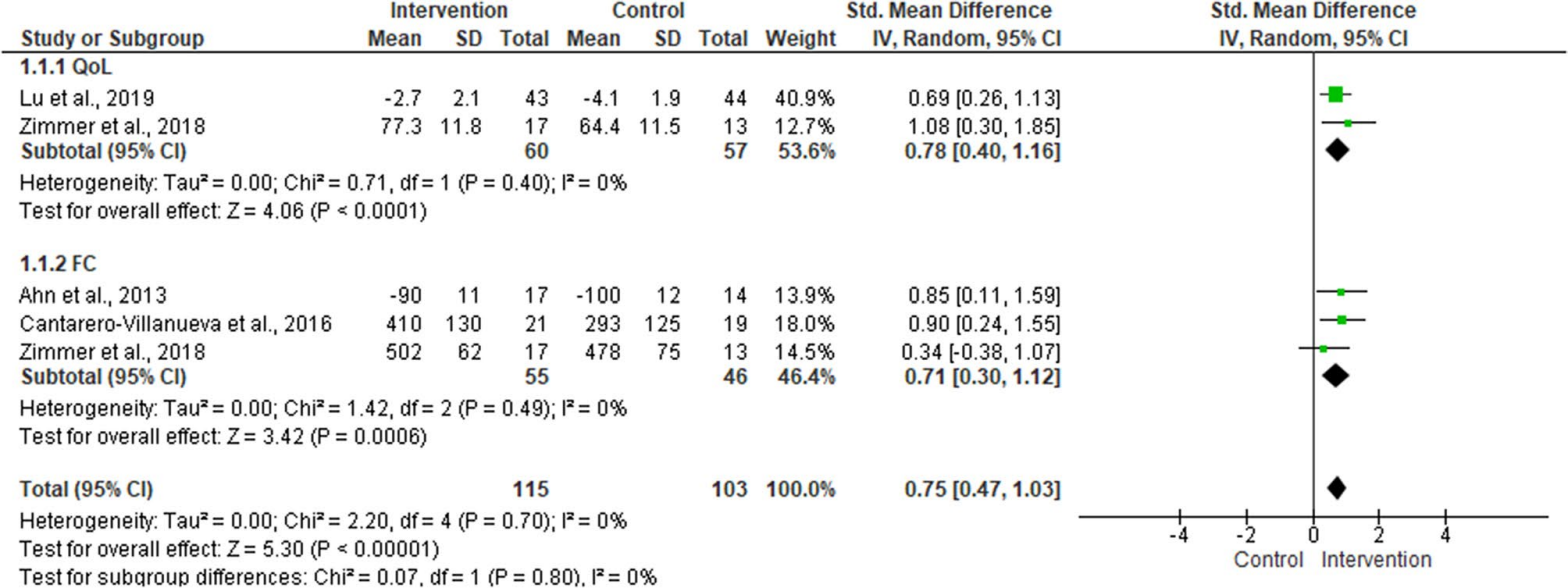
Meta-analysis of quality of life (QoL) and functional capacity (FC) measured in randomized controlled trials that conducted exercise intervention entirely supervised or with some level of supervision (mixed) with ≥ 80 of adherence for colorectal cancer patients.

A meta-analysis revealed a significant effect regarding exercise intervention entirely supervised or with some level of supervision (mixed) for CRC patients (PI − 0.41–1.39) (Fig. 4). The subgroup analysis for the QoL showed a high proportion of variance, near p-value of significance, and a PI of − 6.06–7.16. A considerable I^2^ was obtained for FC with a significant effect and PI of − 0.62–1.52. The adherence to exercise intervention with some level of supervision was 86.3 ± 9.1%. The sensitivity analysis considered four studies^17,33,36,39^ and reinforced the significant effect of intervention with any level of supervision for CRC patients (overall, p = 0.00001, I^2^ = 0%) (Fig. 5).

## Discussion

A meta-analysis revealed that exercise intervention entirely supervised or with any level of supervision is effective in improving the FC in CRC patients. The same outcome was not observed for the QoL. Similarly, no significant effects were observed for home-based interventions in terms of the QoL and FC. However, regardless of exercise intervention supervision, achieving ≥ 80% intervention adherence was associated with improved QoL and FC in CRC patients.

### Modulation of functional capacity and quality of life by home-based exercise intervention

Four home-based interventions evaluated FC and two observed improvements in this outcome^35,42^. The latter interventions adopted aerobic and resistance exercises, but used different instruments for FC measurement. The studies that observed improvements in FC and 83% of the eligible home-based RCTs^32,35,41–43^ provided details in terms of the exercise intensity, which ranged from 64 to 75% of the HR_max_.

This meta-analysis supports the relevance of exercise adherence to improve QoL and FC of CRC patients. The sensitivity analysis revealed that when the adherence was ≥ 80%, the benefits of home-based programs were significant. While Christensen et al.^40^ applied only walking exercises, Lee et al.^42^ and Kim et al.^32^ also allowed hiking, cycling, and resistance exercises. The survivors of ovarian cancer^48^ and CRC^49^ prefer walking as an exercise intervention, and this may have contributed to the improvements in the QoL^32,40^ or FC^42^. Additionally, these studies suggest that gains are observed regardless of the exercises adopted when ≥ 80% of adherence is attained.

Moreover, these interventions lasted 12 weeks, and two^32,42^ detailed the exercise load progression during the training. Although the load progression magnitude was not specified by Christensen et al.^40^, the participants were instructed to perform new tests with the InterWalk application to ensure the exercise program progression. These factors are relevant since the report by Lee et al.^43^ did not observe modifications in the 6-MWT results in a short intervention (6 weeks) and without load progression; however, the physical activity levels and the heart rate after the Tecumseh test were modified in this report. Together, these data highlight the importance of considering training factors such as intervention duration and intensity prescription along with adherence ≥ 80%. The contamination of the control group may explain the non-modification of QoL in the study by Courneya et al.^41^. Pinto et al.^35^ suggested that their small sample size and a possible ceiling effect on the QoL limited the detection of home-based exercise intervention in this outcome.

The eligible studies—except one—evaluated sedentary^35^ patients or patients with low levels of physical activity^32,40,42,43^. Not less important, all eligible home-based RCTs evaluated the CRC patients who underwent surgery or therapy and were mostly non-metastatic. Therefore, future RCTs are required to investigate whether home-based exercise interventions can positively affect the FC and QoL of patients with CRC undergoing ongoing therapy, mainly for those with advanced cancer. This discussion was highlighted in a systematic review by Dittus et al.^50^ in which some eligible studies observed improvements in the QoL, mobility, fatigue, and the sleep quality of stage IV lung cancer and CRC patients^51^ and in fatigue of stage IV breast cancer patients^52^. However, targeted studies are required to define the most appropriate exercise dose according to specific cancer^53^.

### Modulation of functional capacity and quality of life by exercise interventions entirely supervised or with some level of supervision

Both the controls and CRC patients who exercised in the RCT by Lin et al.^44^ improved the FC and QoL. Since the controls were not restricted to physical activity, these authors suggest possible exercise contamination in this group. However, the within-group effect size for the supervised group was moderate to large in some EORTC QLQ-C30 subscales. The FC of CRC patients from the study of Zimmer et al.^17^ did not improve after the intervention. However, among the interventions included in the sensitivity analysis, this study provided the largest SMD on QoL. Other important outcomes, such as the side effects of chemotherapy-induced peripheral neuropathy, balance, and strength were positively modulated. These improvements can be explained by the cautious prescription of the exercise length and intensity (e.g. intensity individualization by heart rate and 1RM). The dose–response of exercise is directly affected by the length of intervention and the precise prescription of the intensity^54^. Therefore, these factors should not be overlooked.

Cantarero-Villanueva et al.^39^ observed improvements of 79.7 m in the FC of CRC patients, while their controls only improved by 4.9 m. These authors emphasized that their exercise program was not designed to improve FC, but health-related parameters including muscle strength. The FC improvements were attributed to gains in abdominal strength, which may have positively influenced the total distance covered in the 6-MWT. Several reports demonstrating the association between muscle strength and FC were brought by the review of Maestroni et al.^55^, strengthening the suggestion of Cantarero-Villanueva et al.^39^. Lu et al.^33^ demonstrated that supervised Baduanjin qigong can decrease the BFI score after 24 weeks of intervention. On the other hand, a recent systematic review and meta-analysis concluded that Baduanjin qigong may have low to moderate efficacy on cancer-related fatigue^56^. Further RCTs involving this practice would confirm its benefits for patients with CRC.

Two mixed interventions were not included in the meta-analysis for those who did not report SD^31^ or those who did not present with exercise adherence^37^. The SMD of the remaining study was favorable to the intervention. Since only one mixed RCT was included in the meta-analysis, further studies with this approach are recommended to verify its effectiveness in improving the QoL and FC in this population.

### Potential advantages and disadvantages of home-based and supervised exercise interventions for patients with CRC

Authors have debated the strengths and limitations of supervised and non-supervised exercise interventions^18–22^. Home-based intervention is a valid strategy to overcome common barriers reported by cancer patients, such as access, time, and cost^57^. However, the home-based definition requires caution interpretation^21^, and proper planning must be conducted even during independent exercise. In line with this, the transition from a supervised environment to a non-supervised was reported as the most significant barrier to exercise by cancer patients^58^. Moreover, a systematic review by Ormel et al.^59^ emphasizes that supervision in a home-based setting can increase exercise adherence by enhancing family support and improving the knowledge and exercise skills of the cancer patient. The latter perspective is aligned with one of the results reported by Hardcastle et al.^60^ in which most of the interviewed non-metropolitan cancer patients were unaware of physical activity recommendations or had incorrect knowledge regarding the intensity required in these guidelines.

One potential advantage of supervised exercise is associated with the control of exercise intensity. The systematic review and meta-analysis of Sweegers et al.^61^ (74 eligible exercise arms) found significant beneficial effects on the QoL and physical function of supervised exercise RCTs in cancer patients. In agreement, non-eligible studies according to our inclusion criteria demonstrated that moderate^62^ and high^15^ intensity supervised exercise promote benefits to cancer patients, including those diagnosed with CRC. Exclusively for CRC patients, the studies suggest high-intensity exercise as a feasible and efficacious intervention for improving the FC^63,64^.

Mixed interventions are feasible^31^ and can reduce the hospital stay after colectomy procedure^36^ and are associated with inhibited global hypermethylation^37^. This approach has emerged as a notable strategy to overcome barriers in home-based or entirely supervised exercise interventions. However, without proper exercise planning, the engagement of the patient, and some experience with exercise, the same limitations previously mentioned for supervised or unsupervised intervention can occur here. Overall, opinion articles have provided an important discussion on the necessity of considering the principles of training and optimizing the safety and efficacy of exercise through cancer treatment^65,66^. Such a perspective must be considered regardless of the supervision level of the exercise intervention.

### Limitations, strengths, and insights for future research

This systematic review and meta-analysis should be interpreted in light of these limitations. Few eligible studies have measured both FC and QoL. None of the mixed interventions have measured the QoL. Moreover, the primary goals of some included RCTs were heterogeneous and the length, frequency, and intensity of the exercise intervention varied substantially. Only 15% of the studies included metastatic patients, and a small sample size was observed in 61% of the RCTs. Furthermore, we cannot discuss the effects of exercise interventions on specific CRC sites (e.g., colon or rectum), which have distinct genotypes and phenotypes, since the included RCTs did not separate the intervention groups according to the tumor location. Future studies should consider this perspective, as CRC types require personalized and individualized treatment^64^. Further research on exercise intervention, regardless of the supervision, for CRC patients must clearly report: (a) the intensity prescription (individualized vs. non-individualized) and the basis for it (i.e., which parameter was adopted to prescribe the intensity); (b) the weekly as well as the total duration; (c) the calculation of load performed during the intervention and how the load progression was performed. If future studies provide efforts to describe these factors, future meta-analyses will be capable of creating subgroups and refining the exercise prescriptions for this population. The strengths of this study are completing the meta-analysis, comparing home-based versus supervised exercise, and performing a novel sensitivity analysis to determine the influence of exercise adherence.

## Conclusion

This systematic review and meta-analysis verified that improvements in the QoL and FC of CRC patients were observed when the adherence to the intervention was ≥ 80%. Such results do not necessarily imply that low physical intervention adherence for this population does not provide any benefits. However, the higher the adherence, the higher are the chances of acquiring benefits in the QoL and FC. Regardless of the supervision characteristics, future RCTs are strongly encouraged to provide a detailed description of exercise variables in physical interventions for CRC prescription. This perspective will allow a refined exercise prescription for patients with CRC, mainly according to their clinical status.
